# Formulation and Evaluation of New Glimepiride Sublingual Tablets

**DOI:** 10.1155/2017/3690473

**Published:** 2017-02-05

**Authors:** Wafa Al-Madhagi, Ahmed Abdulbari Albarakani, Abobakr Khaled Alhag, Zakaria Ahmed Saeed, Nahlah Mansour Noman, Khaldon Mohamed

**Affiliations:** ^1^Department of Pharmaceutical Chemistry, Faculty of Pharmacy, Sana'a University, Sana'a, Yemen; ^2^Department of Pharmacy, Faculty of Medicine and Health Sciences, Yemeni Jordanian University, Sana'a, Yemen; ^3^Department of Pharmacy, Faculty of Medicine and Health Sciences, Thamar University, Dhamar, Yemen; ^4^Department of Biomedical Science, Sana'a University, Sana'a, Yemen

## Abstract

Oral mucosal delivery of drugs promotes rapid absorption and high bioavailability, with a subsequent immediate onset of pharmacological effect. However, many oral mucosal deliveries are compromised by the possibility of the patient swallowing the active substance before it has been released and absorbed locally into the systemic circulation. The aim of this research was to introduce a new glimepiride formula for sublingual administration and rapid drug absorption that can be used in an emergency. The new sublingual formulation was prepared after five trials to prepare the suitable formulation. Two accepted formulations of the new sublingual product were prepared, but one of them with disintegration time of 1.45 min and searching for preferred formulation, the binder, is changed with Flulac and starch slurry to prepare formula with disintegration time of 21 seconds that supports the aim of research to be used in an emergency. The five formulations were done, after adjusting to the binder as Flulac and aerosil with disintegration time of 21 seconds and accepted hardness as well as the weight variation. The assay of a new product (subglimepiride) is 103% which is a promising result, confirming that the formula succeeded. The new product (subglimepiride) is accepted in most quality control tests and it is ready for marketing.

## 1. Introduction

Glimepiride is an orally active hypoglycemic substance belonging to the sulphonylurea group [1, 2]. It acts at ATP-sensitive potassium channels (KATP) on pancreatic *β*-cells to promote insulin release. It binds to 65 kD protein on *β*-cells, which appears to be a part of the same sulphonyl urea receptor that binds Glibenclamide. Glimepiride after oral administration lowers blood glucose 3.5 times more potently than Glibenclamide. Glimepiride is classified under class II according to biopharmaceutical classification system [3]. The drug shows low, pH dependent solubility. In acidic and neutral aqueous media, glimepiride exhibits very poor solubility at 37°C (<0.004 mg/mL). In media with pH > 7, the solubility of drug is slightly increased to 0.02 mg/mL [4]. This poor solubility may cause poor dissolution and unpredicted bioavailability [2, 3]. The very poor aqueous solubility and wettability of glimepiride give rise to difficulties in the design of pharmaceutical formulations and led to variable oral bioavailability so it is beneficial to prepare it into a new dosage form [5]. A novel fast-disintegrating tablet (FDT) can be done based on three-dimensional printing (3DP) technology that can control the material composition, microstructure, and surface texture during its layer-by-layer manufacturing process to provide the products with special properties. In addition, the in vitro release rate can reflect the combined effect of several physical and chemical parameters, including solubility and particle size of the active ingredient and rheological properties of the dosage form. The sublingual (SL) cavity is characterized by unique anatomical and physiological conditions compared with other segments of the GIT such as the stomach and small intestine. A tablet that is swallowed will be subjected to GIT peristalsis in the presence of relatively large volumes of digestive fluids secreted throughout the GIT, facilitating tablet disintegration and drug dissolution. In the SL cavity, tablets are exposed to minimal physiological agitation; moreover, a limited volume of saliva, 0.3 mL/min resting flow rate up to 1 mL/min stimulated flow rate [6], is available to facilitate tablet disintegration and drug dissolution. The sublingual route usually produces a faster onset of action than orally ingested tablets and the portion absorbed through the sublingual blood vessels bypasses the hepatic first-pass metabolic processes [7–10]. Various techniques can be used to formulate rapidly disintegrating or dissolving tablets [11, 12]. This research was aimed at formulating glimepiride into a new fast-disintegrating tablets for sublingual administration as potential emergency to prevent hyperglycemia coma using direct compression technique.

## 2. Material and Methods

### 2.1. Materials

 Glimepiride RS and acetonitrile were obtained from Alpha Chemika, India; sodium dihydrogen phosphate, phosphoric acid, and methanol obtained were from Merck.

### 2.2. Methods

#### 2.2.1. Procedure of Formulation

Sublingual tablets of glimepiride were prepared by the method of direct compression. The excipients used were lactose, pregelatinized starch, sodium starch glycolate, croscarmellose sodium, maize starch, sucralose, and lemon flavor. The accurate amount of the active ingredient and all additives were homogenously blended using geometric dilution after passing through sieve number 60 (standard sieve size) and finally magnesium stearate was added for lubrication and triturated well [13]. Different quantities of excipients were used to prepare various formulations of sublingual tablets as shown in Table 1. The blended material was compressed on 8 mm standard concave punch using a mini press tablet punching machine (RIMEK, India). The total weight of formulation was made up to 150 mg [14, 15].

*Preparation of Formula 1 (F1)*. All ingredients were weighed separately and then mixed except for Mg-stearate and glimepiride by geometric mixing. Finally the Mg-stearate was added to the final mixture and compressed. 

*Preparation of Formula 2 (F2)*. All ingredients were weighed separately and then mixed together except for Mg-stearate and glimepiride by geometric mixing. After that, he glimepiride was added to mixture according to geometric mixing. The mixture was granulated by distilled water and dried in oven at 50°C.

After drying, the mixture was passed through sieve (0.5 mm). Finally, the Mg-stearate was added to the final mixture and compressed.

*Preparation of Formula 3 (F3)*. All ingredients were weighed separately. The pregelatinized starch was mixed with distilled water until a paste was formed. Then, the glimepiride, maize starch, 2/3 sodium starch glycolate, and 2/3 croscarmellose sodium were mixed together and then the mixture was granulated by paste and dried in oven 50°C. The granulated mixture passed through sieve 35 (0.5 mm). After that, 1/3 sodium starch glycolate and 1/3 croscarmellose sodium were added to mixture and mixed. Finally, the Mg-stearate was added to the final mixture and compressed.

*Preparation of Formula 4 (F4)*. All ingredients were weighed separately and then the pregelatinized starch was mixed with distilled water until a paste was formed. After that, the glimepiride, maize starch, 2/3 sodium starch glycolate, 2/3 croscarmellose sodium, and povidone were mixed together and then the mixture was granulated by paste and dried in oven at 50°C. After that, the mixture granules were sieved by sieve 35 (0.5 mm). Then, 1/3 sodium starch glycolate and 1/3 croscarmellose sodium were added to the mixture and mixed. Finally, the Mg-stearate was added to the final mixture and compressed.

*Preparation of Formula 5 (F5)*. All ingredients were weighed separately. The glimepiride, maize starch, aerosil 200, and dicalcium phosphate were mixed together. After binder was prepared (starch paste), the mixture was granulated by starch paste and dried at 45°C. After that, the aerosil and Flulac were mixed; then sucralose and lemon flavor were added to this mixture. Finally, the Mg-stearate was added and compressed.

*Preparation of Starch Paste (the Binder)*. The maize starch was dissolved in 3 L of distilled water (40–45°C) and was checked for being free of lumps. This slurry was charged into 10 mL of water (95°C) into vessels until complete gelatinization. Finally, the mixture cooled to 50°C.

#### 2.2.2. Procedure of Evaluation of the Best Formula

(*1) Chemical Test*. Assay test was done using HPLC method as follows.

*Mobile Phase Preparation*. Sodium di-hydrogen phosphate 0.5 gm was dissolved in 500 mL of distilled water. Adjust pH to 2.4 with H_3_PO_4_ and add 500 mL of acetonitrile; mix it well and filter it through 0.45 *μ*m micromembrane filter.

*HPLC Conditions*. The conditions were as follows: column: ODS1 (C18) 15*∗*0.45 cm; flow rate: 1.0 mL/min; wavelength: 220 nm; sensitivity: 1; pressure: 28 Mpa.

*Preparation of Standard Solution*. Weigh accurately equivalent to 21 mg of glimepiride RS into 100 VF and dissolve and then dilute it with acetonitrile solution 80% and mix well to get concentration at 0.21 mg/mL.

*Preparation of Sample Solution*. Transfer 7 tablets (equivalent to 21 mg of glimepiride) into 100 VF and dilute them to with acetonitrile solution 80% and mix well to get concentration. Sonicate the solution for 10 minutes and filter to get 0.21 mg/mL [16].

(*2) Physical Test: Micrometrics*. The thickness and diameter of 10 tablets were measured using micrometres. Limits: diameter should be less than 13 mm.

*Weight Variation*. 20 tablets of the product were weighed; then the higher limit (HL) and lower limit (LL) were calculated as follows:

Average  wt = total  wt/20
Average  wt × 5% = *n*
HL = Av.  wt + *n*
LL = Av.  wt − *n* [16, 17]

*Friability Test*. The tablets of this product were weighed (*w*1), then put in the instrument for 4 minutes, and then weighed again (*w*2). After that, the following were calculated:

Friability = [(*w*2)/(*w*1)]*∗*100
Limit for compression tabs: not more than 1% [16]

*Hardness Test*. Ten tablets were put in specific place and fixed; then turn it on and wait until the fraction occurs. The limit is 4–8 kg/cm^2^ [16].

*Disintegration Time Test*. The disintegration time is calculated using disintegrator using water as media and the limit of tablet is 5–30 minutes [16].

### 2.3. Statistical Analysis

Data were presented as means ± standard deviation (SD). SPSS version 12 was used for statistical analysis. A *t*-test and the one-way ANOVA were performed to examine the differences among the groups. A *P* value of <0.05 was considered to be statistically significant.

## 3. Results and Discussion

In formula 1 (F1), the tablets were very friable and cannot be compressed because the hardness was weak due to the presence of high amount of croscarmellose as disintegrant agent and, therefore, the binder would increase in formula 2. Formula 2 (F2) showed disintegration time of about 1.45 min, but while searching for a preferred formula with fast disintegration, low croscarmellose sodium was used as disintegrant agent and therefore add super disintegrant, flavoring agent, and sweetening agent and change the diluent in formula 3. Formula 3 (F3) showed the high friability of tablet due to the fact that the binder is not suitable and, therefore, add new binder in formula 4. In formula 4 (F4), the hardness is strong due to the fact that the binder is high and not suitable and therefore change the formula. Formula 5 showed the best result as shown in Table 2.

The new sublingual formula showed good disintegration with 21 seconds which is more preferred than formula 2, as the assay gives good result compared to Amaryl as the standard drug as well as the hardness and friability.

## 4. Conclusion

The five formulations were done and after adjusting to the binder as Flulac and aerosil with disintegration time of 21 seconds and accepted hardness as well as the weight variation. The assay of new sublingual glimepiride is 103% which is a promising result and confirms that the formula succeeded and is accepted in most quality control tests and it is ready for marketing. The sublingual glimepiride has fast dissolving time that would be more targeted in emergency DM.

## Figures and Tables

**Table 1:** The table illustrates the materials and their quantities which used to formulate new glymipride subligual tablet formulations (F1, F2, F3, F4, and F5).

Number	Material	Formula code									
		F1		F2		F3		F4		F5	
		Amount/unit	Actual amount	Amount/unit	Actual amount	Amount/unit	Actual amount	Amount/unit	Actual amount	Amount/unit	Actual amount
1	Glimepiride	0.003 g	0.9 g	0.003 g	0.9 g	0.002 g	0.6 g	0.003 g	0.9 g	3 mg	0.9 g
2	Lactose	0.05 g	16.5 g	0.069 g	20.7 g	—	—	—	—	—	—
3	Croscarmellose sodium	0.01 g	3 g	0.026 g	7.8 g	—	—	—	—	—	—
4	Pregelatinized starch	0.02 g	6 g	0.02 g	6 g	0.002 g	0.6 g	0.0033 g	1 g	—	—
5	Mg-stearate	0.002 g	0.6 g	0.002 g	0.6 g	0.002 g	0.6 g	0.002 g	0.6 g	0.5 mg	0.15 g
6	Mais starch	—	—	—	—	0.002 g	0.6 g	0.06 g	18 g	61 mg	18.3 g
7	Na starch glycolate	—	—	—	—	0.002 g	0.6 g	0.02 g	6 g	—	—
8	Croscarmellose Na	—	—	—	—	0.002 g	0.6 g	0.02 g	6 g	—	—
9	Sucralose	—	—	—	—	0.002 g	0.6 g	0.003 g	0.9 g	3 mg	0.9 g
10	Lemon flavor	—	—	—	—	0.002 g	0.6 g	0.002 g	0.6 g	2 mg	0.6 g
11	Povidone	—	—	—	—	—	—	0.002 g	0.6 g	—	—
12	Aerosil 200	—	—	—	—	—	—	—	—	1 mg	0.3 g
13	Aerosil	—	—	—	—	—	—	—	—	1 mg	0.3 g
14	Flulac	—	—	—	—	—	—	—	—	10 mg	3 g
15	Dicalcium phosphate	—	—	—	—	—	—	—	—	40 mg	12 g

Total		0.09 g	27 g	0.12 g	36 g	0.1133 g	34 g	0.1153 g	34.6 g	107.5 mg	36.45 g

**Table 2:** The table shows the reading and results of glimepiride sublingual tablet formulation.

Name of the test	Reading				Comments
	Amaryl		F5 (new glimepiride)		
Assay	100.5%		103%		Conformed

Friability	0.02%		0.40%		Conformed

Weight variation	1	0.1669	0.116		Conformed
	2	0.168	0.116		
	3	0.1676	0.116		
	4	0.167	0.115		
	5	0.1687	0.115		
	6	0.1673	0.115		
	7	0.168	0.113		
	8	0.1688	0.115		
	9	0.1679	0.114		
	10	0.169	0.114		
	11	0.1672	0.113		
	12	0.169	0.115		
	13	0.1673	0.117		
	14	0.1684	0.117		
	15	0.169	0.115		
	16	0.1685	0.111		
	17	0.1669	0.111		
	18	0.1674	0.111		
	19	0.1678	0.115		
	20	0.1678	0.114		
	Average	0.167925	0.1141		

	Number	Reading	Number	Reading	

Hardness	1	5.35	1	3.8	Not conformed
	2	4.28	2	4.5	Conformed
	3	4.06	3	4.3	Conformed
	4	3.81	4	4.3	Conformed
	5	5.3	5	3.5	Not conformed

Disintegration time	1.15 minutes		21 seconds		Conformed
